# Exploring the Comparative Efficacy of Metformin and Resveratrol in the Management of Diabetes-Associated Complications: A Systematic Review of Preclinical Studies

**DOI:** 10.3390/nu12030739

**Published:** 2020-03-11

**Authors:** Phiwayinkosi V. Dludla, Sonia Silvestri, Patrick Orlando, Kwazi B. Gabuza, Sithandiwe E. Mazibuko-Mbeje, Tawanda M. Nyambuya, Vuyolwethu Mxinwa, Kabelo Mokgalaboni, Rabia Johnson, Christo J. F. Muller, Luca Tiano, Johan Louw, Bongani B. Nkambule

**Affiliations:** 1 Biomedical Research and Innovation Platform, South African Medical Research Council, Tygerberg 7505, South Africa; kwazi.gabuza@mrc.ac.za (K.B.G.); rabia.johnson@mrc.ac.za (R.J.); johan.louw@mrc.ac.za (J.L.); 2 Department of Life and Environmental Sciences, Polytechnic University of Marche, 60131 Ancona, Italy; s.silvestri@univpm.it (S.S.); p.orlando@univpm.it (P.O.); l.tiano@univpm.it (L.T.); 3 Division of Medical Physiology, Faculty of Health Sciences, Stellenbosch University, Private Bag X1, Tygerberg 7505, South Africa; sithandiwe.mazibukombeje@gmail.com; 4 Department of Biochemistry, Faculty of Natural and Agricultural Sciences, North-West University, Mmabatho 2745, South Africa; 5 School of Laboratory Medicine and Medical Sciences, College of Health Sciences, University of KwaZulu-Natal, Durban 4000, South Africa; mnyambuya@nust.na (T.M.N.); 218081787@stu.ukzn.ac.za (V.M.); 218086707@stu.ukzn.ac.za (K.M.); nkambuleb@ukzn.ac.za (B.B.N.); 6 Department of Health Sciences, Faculty of Health and Applied Sciences, Namibia University of Science and Technology, Windhoek 9000, Namibia; 7 Department of Biochemistry and Microbiology, University of Zululand, KwaDlangezwa 3880, South Africa

**Keywords:** metformin, resveratrol, combination therapy, diabetes mellitus, dietary supplements, antioxidants

## Abstract

Food-derived bioactive compounds such as resveratrol are increasingly explored for their protective effects against metabolic complications. Evidence supports the strong antioxidant properties and therapeutic effects of resveratrol in managing diabetes and its associated complications. However, evidence informing on the comparative or combination effects of this natural compound with an accomplished and well-characterized antidiabetic agent like metformin has not been revised. Thus, we conducted a comprehensive systematic search of the major electronic databases which included MEDLINE, Cochrane Library, and EMBASE. The cumulative evidence strongly supports the comparative effects of metformin and resveratrol in ameliorating diabetes-associated complications in preclinical settings. In particular, both compounds showed strong ameliorative effects against hyperglycemia, dyslipidemia, insulin resistance, a pro-inflammatory response, and lipid peroxidation in various experimental models of diabetes. Enhancing intracellular antioxidant capacity in addition to activating NAD-dependent deacetylase sirtuin-1 (SIRT1) and AMP-activated protein kinase (AMPK) are the prime mechanisms involved in the therapeutic effects of these compounds. Of interest, preclinical evidence also demonstrates that the combination treatment with these compounds may have a greater efficacy in protecting against diabetes. Thus, confirmation of such evidence in well-organized clinical trials remains crucial to uncover novel therapeutic strategies to manage diabetes and its linked complications.

## 1. Introduction

It has been centuries since diabetes mellitus was first described [1], however, this metabolic condition continues to greatly impact the quality of life of patients living with diabetes [2,3]. Besides being one of the top ten causes of death worldwide [4], patients with diabetes are at an increased risk of comorbidity of complications associated with metabolic disorders such as neuropathy, retinopathy, and cardiovascular diseases (CVDs). These comorbidities significantly reduce the quality of life, and may even accelerate the rate of permanent disability [5,6]. Diabetes is often described in terms of its impact on working-age adults, however, it is of concern that even in older adults (≥60 years old), this condition accelerates the rate of mortality, while also enhancing the risk of institutionalization [7]. Rapid urbanization that is frequently accompanied by a sedentary lifestyle, mostly identified in developed and developing countries, has notably been responsible for the global rise in cases of type 2 diabetes (T2D) [8,9]. T2D is associated with obesity and is a predominant form of diabetes that is characterized by hyperglycemia, insulin resistance and dyslipidemia. Although the pathological mechanisms implicated in the development of diabetes have been partially described [10,11,12], long-term effective therapeutic strategies to contain this metabolic disorder remain scarce. 

Several studies have demonstrated that improving blood glucose control can revert diabetes-associated complications, especially by complementing antidiabetic drugs with lifestyle modification intervention, such as regular physical activity [13,14,15]. However, the major challenge has been the limitation of evidence showing the long-term effectiveness of antidiabetic drugs, this coupled with so few individuals that can maintain consistent physical activity could explain the continued rise in new cases of diabetes over the past decades [2,3]. Alternatively, there has been great interest in exploring nutritional food products, including plant derived-bioactive compounds, for their ameliorative effects against diabetes-associated complications. For example, regular intake of tea or fruits, due to their rich source of several nutrients and phytochemicals, has been associated with reduced risk of several chronic diseases, including obesity and diabetes [16,17,18]. Consistently, evidence from a meta-analysis of randomized controlled trials (RCTs) has shown that supplementation with resveratrol could effectively improve fasting plasma glucose and insulin levels [19]. Moreover, the abundant antioxidant properties of resveratrol [20] further promote its therapeutic value, especially its use as a dietary supplement to manage diabetes-associated complications. 

Resveratrol (PubChem CID: 445154) can be found in several food sources including grapes and red wine [21]. Interestingly, experimental evidence reporting on the comparative or combination use of metformin (PubChem CID: 4091) with well-known bioactive compounds like resveratrol has become of great interest [22,23,24,25,26,27]. Although metformin is a commonly used antidiabetic drug, information on its comparative or combination use with resveratrol has not been assessed to understand the potential synergistic or combination advantages of such a therapy in managing metabolic complications. Thus, the current systematic review aimed to provide a comprehensive synthesis of preclinical studies assessing the comparative effects of metformin with resveratrol against diabetes-associated complications. Information related to the more bioactive compound of the two, as well as evidence linked to adverse or beneficial effects if both compounds are combined is discussed.

## 2. Materials and Methods 

The Preferred Reporting Items for Systematic reviews and Meta-Analysis (PRISMA) guidelines were followed to prepare the current systematic review [28]. Uploaded file 1 illustrates the PRISMA checklist for this systematic review. Moreover, the International prospective register of systematic reviews (PROSPERO) was carefully examined to make sure there is no registered systematic review that is investigating a similar topic.

### 2.1. Search Strategy

For study inclusion, major electronic databases such as MEDLINE, Cochrane Library, and EMBASE were searched from inception up to 30 November 2019. Briefly, two reviewers (PVD and TMN) independently conducted this process, while a third commentator (BBN) was referred for adjudication. The search strategy was adapted to the respective database using keywords and medical subjects heading (MeSH) terms such as “metformin,” “resveratrol,” “diabetes mellitus,” “metabolic syndrome,” “hyperglycemia,” and their corresponding synonyms and associated words/phrases. EndNote version 10 (Clarivate Analytics, Philadelphia, USA) was used to manage extracted information, also to remove any duplicates, as previously reported [29]. No language restrictions were applied. 

### 2.2. Inclusion and Exclusion Criteria

The primary search encompassed all experimental studies reporting on the impact of metformin, in comparison or when combined with resveratrol, against diabetes or its related complications. In vitro studies included those examining the direct effect of treatment/intervention on experimental models of cultured immortal or primary isolated cells from rodents or human subjects with diabetes or metabolic syndrome. In vivo studies were on isolated cells from treated diabetic rodents or humans. Ongoing studies, as well as review articles, were screened for primary findings, while editorials, books and letters were excluded.

### 2.3. Data Extraction and Assessment of Quality 

Two investigators (PVD and TMN) separately assessed all relevant articles and carefully chose those that were relevant. Disagreements regarding the study selection were resolved by referring to a third investigator (BBN). The primary outcome of the study was to compare the ameliorative effects of metformin with that of resveratrol in ameliorating diabetes-associated complications. The secondary outcome was to establish whether resveratrol improves the ameliorative effects of metformin against diabetes-associated complications. To achieve this, relevant data items, from each article were extracted. These included author name and year of publication, the experimental model used, the doses of treatment compounds and intervention period, and the main findings. Moreover, two investigators, TMN and KM, made use of Animal Research: Reporting In Vivo Experiments (ARRIVE) guidelines to assess nonhuman studies, as per a previously published protocol [30]. Any discrepancies were resolved by consulting the third investigator (VM). 

## 3. Results

### 3.1. Characteristic Features of Included Studies 

**Figure 1:**
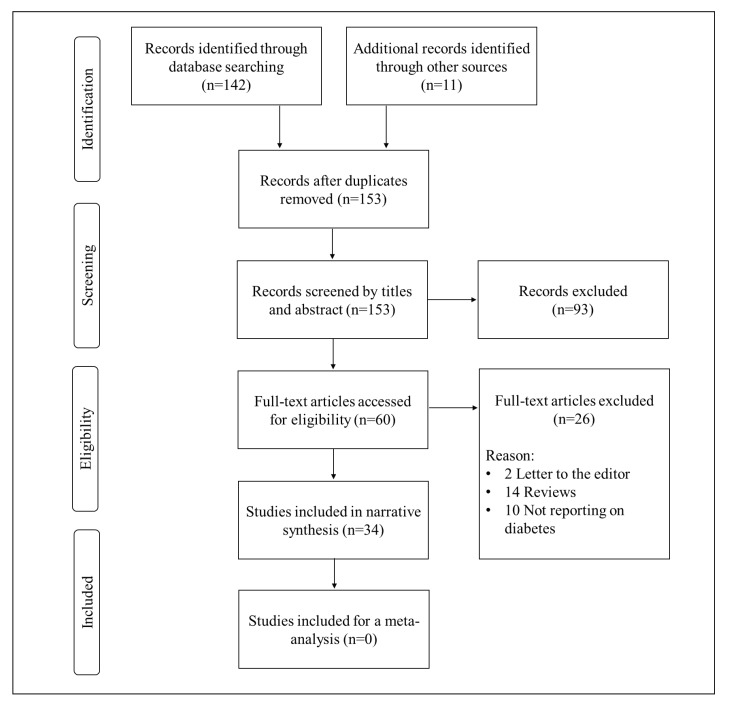
A flow diagram showing the study selection and inclusion criteria.

A total of 153 records were acquired through the combined systematic search of the literature. The comprehensive flow diagram of included and excluded studies is represented in Figure 1. While 93 studies were initially excluded for being relevant, as much as 26 studies were disregarded because they were reviews, letters to the editor or they did not report on diabetes. Subsequently, 34 documents involving preclinical studies were included and are comprehensively discussed below. 

### 3.2. Risk of Bias and Quality of the Studies

The quality of included studies was assessed by TMN and VM, using the ARRIVE guidelines [30]. All included animal studies in this systematic review met the minimum requirements for publication using the ARRIVE guidelines checklists with 20 questions. Briefly, the median score range of all included studies was 15 (10–19) out of a possible score of 20. The introduction domain had a median score of 4 (4-4) out of the possible score of 4 (overall agreement 100%, kappa = 1) whilst the methods domain had a median score of 6 (2-9) out of a possible score of 9 (overall agreement 88.89%, kappa = 0.76). Moreover, the studies scored high in the results and discussion domains with a median score of 2 (1-3) out of the possible score of 4 (overall agreement 87.50%, kappa = 0.75) and 3 (2-3) out of the possible score of 3 (overall agreement 91.67%, kappa = 0.84), respectively.

### 3.3. In vitro Evidence on the Comparative Effects of Metformin with Resveratrol Against Diabetes-Associated Complications

It is well-established that diabetes can affect several organs of the body, leading to the development of diverse pathological conditions, including neuropathy, retinopathy, nephropathy, and CVDs [2,3]. Currently, various experimental models exist that are used to understand the pathophysiology of the aforementioned pathologies. Most importantly, these are used to test various pharmacological compounds for their ameliorative effects against such complications using these experimental models [31]. In fact, in vitro cell- and tissue-based systems have become more relevant for routine screening of bioactive compounds for their ameliorative properties against different disease conditions, especially during the early stages of drug development [32]. Table 1 provides an overview of studies reporting on the comparative effect of metformin and resveratrol against diabetes-associated complications using in vitro models. In addition to specifying the experimental model used, summarized evidence included displaying the precise dose for each treatment compound, as well as the relevant intervention period. 

Briefly, human umbilical vascular endothelial cells (HUVECs), 3T3-L1 cells and C2C12 myotubes were some of the experimental models used to investigate the comparative effects of metformin to resveratrol in controlling diabetes-linked abnormalities in vitro [33,34,35,36,37,38,39,40,41]. The cumulative evidence supports the comparative effects of metformin and resveratrol in attenuating high-glucose-induced lipid overload and cell apoptosis [33,34,37,39,42], improving β-cell function [34] and preventing hypoxia by limiting oxygen consumption [39]. In addition to activating NAD-dependent deacetylase sirtuin-1 (SIRT1) and AMP-activated protein kinase (AMPK), metformin and resveratrol ameliorated high glucose-induced damage and lipid overload, improved glucose transport, reduced reactive oxygen species (ROS) and attenuated pro-inflammatory/apoptotic markers such as nuclear factor kappa-light-chain-enhancer of activated B cells (NF-κB) and bcl-2-like protein 4 (BAX) expression [33,34,35,36,37,38,39,40,41]. Interestingly, similar to the accomplished capacity of resveratrol in cellular regulation via activating SIRT1, an in silico study [43] showed that metformin can interact with the very same allosteric site occupied by resveratrol and other sirtuin-activating compounds (STATCs) at the amino-terminal activation domain of SIRT1. In a similar study, enzymatic assays confirmed that the net biochemical effect of metformin and other biguanides such as a phenformin was to improve the catalytic efficiency of SIRT1 operating in conditions of low NAD+ in vitro. Thus, such findings could explain the comparative effects observed with the use of metformin and resveratrol in vitro.

Only a few studies reported on the negative effects of metformin and resveratrol in vitro. For instance, one study showed that these compounds failed to stabilize tubes and or enhance the paracrine angiogenic activity of human myeloid angiogenic cells [44]. Prolonged treatment with metformin could show better effect than resveratrol in preventing senescent by modulating SIRT1/p300/p53/p21 pathway in HUVECs were exposed to high glucose [38]. Beyond reporting on the comparative impact of metformin and resveratrol in controlling high glucose (hyperglycemia)-induced associated complications, other studies also assessed the modulatory effect of these compounds on crucial cytochromes P450 (CYPs) enzymes. In brief, most of these enzymes are known to be located in the inner-mitochondria and the endoplasmic reticulum, and in addition to being involved in drug metabolism, some of their function is associated with cholesterol and lipid synthesis [46]. Consistently, metformin and resveratrol were shown to be able to block protein expression and enzyme activities of these cytochrome enzymes (CYP17 and CYP21) [41]. However, only SIRT3 mRNA expression appeared to be transformed by resveratrol, but SIRT1, 3 and 5 overexpression did not result in alteration in the steroid profile of H295R cells [41]. Findings from this study further showed that resveratrol could block the steroidogenesis of rat ovarian theca cells by inhibiting the protein kinase B (PKB/Akt) pathway. Further suggesting that resveratrol could be a candidate drug for the treatment of hyperandrogenic disorders such as polycystic ovary syndrome. However, this requires further exploration in other experimental models, also looking at a broader network of molecular mechanisms involved. 

### 3.4. In Vivo Evidence on the Comparative Effects of Metformin with Resveratrol Against Diabetes-Associated Complications

Impaired insulin signaling is associated with the development of insulin resistance, obesity, and hyperglycemia, which are a hallmark of T2D [8,9]. On the other hand, type 1 diabetes (T1D), which is also known as juvenile diabetes, may occur as a result of autoimmune destruction of insulin-producing pancreatic β-cells [8,9]. While a high-fat diet (HFD) is an established model to induce T2D in rodents, streptozotocin (STZ), a naturally occurring alkylating antineoplastic agent, has also been widely used to render T1D in various animal models [47]. Other studies have used the combination of a low dose of STZ and HFD to mimic conditions of T2D [47]. Alternatively, Zucker Diabetic Fatty rats, Goto-Kakizaki rats, Akita mice model, and db/db been genetically modified rodent models that have become even more relevant spontaneous development of metabolic complications associated with T2D [47]. Table 2 provides an overview of studies reporting on the comparative effects of metformin versus resveratrol against diabetes-linked abnormalities through the use of various in vivo animal models. 

Several studies assessed the comparative effects of metformin and resveratrol through the use of chemicals such as STZ and alloxan to induce diabetes-associated complications (Table 2). In brief, Chi and colleagues demonstrated that resveratrol produces a similar effect to metformin in reducing elevated plasma glucose levels while increasing insulin levels in STZ-induced diabetic Wistar rats [48]. This was followed by normalization of hepatic phosphoenolpyruvate carboxykinase (PEPCK) expression, activating phosphatidyl-3-kinase (PI3K), and increased glucose transporter (GLUT) 4 levels in the soleus muscle of STZ-diabetic rats. Other studies consistently reported on comparative outcomes with the use of both compounds in improving metabolic profiles, ameliorating hepatic and pancreatic β-cell damage, and blocking oxidative stress [25,52]. Attenuation of ROS production, with activation of AMPK, were the predominant mechanism associated with the activity of metformin and resveratrol Interestingly, in alloxan-induced diabetic rats, supplementation with resveratrol alone or in combination with vitamin E for 30 days provided a better effect than metformin in ameliorating the hyperglycemia-induced complications [57]. Thus, suggesting that resveratrol could offer synergistic effects in ameliorating diabetes-associated complications when used in combination with other antioxidant compounds. However, these findings need further exploration using other experimental models.

Table 2 also showed that fructose-rich (HFD diet)-induced metabolic alterations was the predominant model used in most studies that assessed the comparative effects of metformin with resveratrol in vivo. Through the exploration of this model, it was demonstrated that both metformin and resveratrol could effectively revert adipose tissue dysregulations and improve endothelium-dependent vasodilation [26,42,44,50,55], concomitant to protecting against inflammation-induced defects [25,39,51], and enhance heart function [54]. Some of the prime mechanisms associated with the effectivity of these compounds included activating AMPK, reducing free fatty acid influx and diacylglycerol accumulation, blocking protein kinase C activity, reducing tumor necrosis (TNF)-α/interleukin (IL)-6/ interferon-gamma levels, and decreasing lipid peroxidation. 

Interestingly, while the comparative effect of these compounds was acknowledged in vivo, some of the reported evidence displayed an improved effect of resveratrol when compared to metformin in ameliorating diabetes-associated complications. For example, although both compounds could normalize altered metabolic parameters, resveratrol showed more potency in improving insulin sensitivity and attenuating oxidative stress parameters in fructose-fed male Sprague Dawley rats [49,53]. This was concomitant with the capability of this compound to significantly reduce thiobarbituric acid reactive substances levels by improving intracellular antioxidants such as decreased superoxide dismutase (SOD) in addition to promoting the activation of antioxidant response mechanisms through the upregulation of nuclear factor erythroid 2-related factor 2. Other studies also demonstrated that while metformin could slow the rate of muscle fiber aging, resveratrol provided a better effect in decelerating the aging of neuromuscular junctions (NMJs) in the extensor digitorum longus muscle of 2-year-old mice fed 40% less fat calories [56]. 

### 3.5. Experimental Evidence on the Combined Effects of Metformin with Resveratrol Against Diabetes-Associated Complications

Consistent with already summarized information on the comparative effects of metformin with resveratrol, emerging data have also explored the combination use of these compounds in various preclinical models of diabetes. Table 3 gives an overview of studies reporting on the combined effect of metformin with resveratrol against diabetes-associated complications, through the use of various pre-clinical models. 

In vitro evidence, on cultured skeletal muscle cells (C2C12) and adipocytes (3T3-L1), when used together with hydroxymethylbutyrate showed that the combination of metformin with resveratrol was more effective at increasing fat oxidation than the use of each compound as a monotherapy [58]. The prominent mechanisms implicated in this process included the activation of AMPK and SIRT1. Interestingly, the same study supported the superior effect of combination therapy in improving insulin sensitivity, plasma insulin levels, and insulin tolerance test response in type 2 diabetic (db/db) mice [58]. In a similar experimental model [22], it has been demonstrated that the combined treatment could reduce obesity, glucose, and triglyceride levels, as well as improve renal function a liver function. 

Confirming results from a db/db mouse model [58], using HFD-fed mice the combination of metformin and resveratrol treatment for 5 weeks was shown to be more effective than the use of metformin as a monotherapy in improving glucose tolerance [59]. Such findings were replicated by others demonstrating that combination treatment could reduce body and ovary weights concomitant to improving blood glucose control and ameliorating inflammation in HFD-fed rodents [60,61,62]. In addition to activating SIRT1, AMPK and raptor (the regulatory subunit of mTORC1), mechanisms associated with the beneficial effect of combination treatment included decreasing luteinizing hormone, follicle-stimulating hormone, TNF-α, and tissue anti-mullerian hormone levels, also blocking lipid peroxidation by reducing increased malondialdehyde. Interestingly, while Frendo-Cumbo and coworkers [23] demonstrated that combination treatment for 4 weeks was more effective than the use of each compound alone in improving glucose and insulin tolerance, combination treatment did not affect body weight, adiposity, or markers of adipose tissue inflammation in these mice. Although speculative, the use of a relatively higher dose of resveratrol in this study [23], when compared to other findings on similar [60,61,62], could explain the failed effect of combination therapy in impacting body weight. However, additional studies are required for exploring the dose and time-dependent impact of combination treatment in various experimental models of diabetes. 

## 4. Discussion

Noncommunicable diseases remain the leading cause of death worldwide [2,3]. Moreover, metabolic-related complications are known to play a major role in accelerating the global mortality rate [2,5,6]. Thus, highlighting the importance and the need to urgently establish novel and effective therapies to contain metabolic disorders. Metformin is widely considered as a drug of choice in the treatment of T2D [63], however, the long-term use of this drug seems to be less effective to contain diabetes-associated complications, as evident by the rapidly rising prevalence of diabetes [2,3]. As a result, alternative strategies have been progressively explored to contain diabetes and its linked complications. 

For example, there is a need to understand the beneficial effects of metformin against diabetes-associated complications when used in combination with other antidiabetic drugs like vildagliptin or sulphonylurea [64,65]. Similarly, due to the great interest in understanding the bioactive properties of various naturally-derived compounds such as resveratrol, its combination uses with established oral glucose-lowering drugs like metformin could be of therapeutic benefit to combat diabetes. In fact, in many experimental settings, metformin is widely used as an experimental control to compare the efficacy of other pharmacological compounds for their antidiabetic properties [33,34,35,36,37,38,39,40,41]. To our knowledge, this is the first systematic review to assess and synthesize available evidence reporting on the comparative or combined effects of metformin with resveratrol for their ameliorative effects against diabetes and its associated complications. This is especially important to establish whether resveratrol possesses similar effects to metformin or find out whether this naturally-derived compound can improve the efficacy of metformin in protecting against diabetes.

A systematic search of literature retrieved approximately 34 studies informing on essential information related to the comparative or combination effect of metformin and resveratrol. Significant findings on the comparative use of metformin with resveratrol verified that both compounds possess comparable biological properties in attenuating diabetes-associated complications in vitro [33,34,37,39,42]. As displayed in Table 1, the use of human peripheral blood CD34+ cells or human adrenal H295R cells, consistent with 3T3-L1 adipocytes, HepG2 hepatocytes, and HUVECs as well as INS-1E beta-cells were some of the in vitro models employed to assess the comparative effects of metformin in ameliorating diabetes-related abnormalities. Here, the overall findings support the comparative effects of metformin and resveratrol in reverting β-cell, cardiac, and hepatic damage [34], preventing hypoxia-induced cellular injury [39]. It was also evident that high-glucose exposure was the predominant means to explore hyperglycemia-induced stress. Consistently, activation of SIRT1 and AMPK, together with a reduction of intracellular lipid overload, improvement of glucose transport, amelioration of ROS, and attenuation of pro-inflammatory/apoptotic markers such as NF-κB and BAX expression were the predominant mechanism by which metformin and resveratrol could control diabetes-associated complications in vitro [33,34,35,36,37,38,39,40,41]. 

While strong antioxidant properties and hypoglycemic effects could be attributed to the beneficial effects of metformin and resveratrol [20,23,38,62,66], an interesting finding from an in silico study [43] demonstrated that both compounds could share a similar activity by interacting with SIRT1. Fascinatingly, SIRT1 together with AMPK are largely involved in the regulation of many cellular processes, including energy metabolism, cell cycle, insulin secretion, and apoptosis during the pathogenesis of diabetes [67,68,69,70]. Thus, the capability of both metformin and resveratrol to comparable modulate these molecules suggests their strong aptitude to control diabetes-complications. Nevertheless, such evidence was confirmed in vivo [26,42,44,50,55], with data supporting the strong potential for both compounds to attenuate diabetes-linked abnormalities equally in STZ or HFD-fed induced models, representing T1D and T2D, respectively. Moreover, some of the data demonstrated that resveratrol might be more protective than metformin in vivo [54,56,57], however, these are only preliminary findings this aspect needs further exploration using well-designed experiments.

In relation to the combination use of metformin with resveratrol, cumulative evidence shows that the combined use of these compounds consistently ameliorates diabetes-associated complications in vitro and in vivo, as displayed in Table 3. In particular, the combined treatment showed better efficacy in ameliorating oxidative stress, than the use of metformin or resveratrol as a monotherapy. Additionally, the combined treatment attenuated inflammation, while also showing enhanced effect in improving glucose tolerance via activation of the insulin-dependent mechanisms such as the PI3K/protein kinase B (AKT) pathway [23,58,60]. Interestingly, in adult patients on metformin therapy, the use of resveratrol is equally effective in reducing body weight, improving glucose tolerance, and also improving CVD-related outcomes by lowering systolic blood pressure, total cholesterol, triglyceride, urea nitrogen, and total protein [71,72]. Although some results showed it could not affect body weight, arterial blood pressure or fasting plasma glucose [73,74], supplementation of resveratrol in patients on metformin was still beneficial in improving antioxidant enzymes such as SOD, catalase, reduced GSH levels, while also reducing lipid peroxidation [73,74]. Therefore, implying that the combined use of metformin with dietary supplements with glucose-lowering and strong antioxidant properties could be of benefit in containing diabetes-related complications, and is worth further exploration.

In addition to a broadly dissimilar dose selection between reported findings, the evidence presented in this review demonstrates that the duration of treatment with either metformin or resveratrol also varied across in vivo experimental models of diabetes or metabolic syndrome (Table 2). For instance, although attenuation of diabetes-associated complications was evident as early as seven days of treatment in rodents [39,48], constant results were observed with prolonged intervention period lasting up to eight weeks [49,50,53]. Similarly, when the compounds were used in combination, the beneficial effects were observed with the intervention period between four to six weeks (Table 3), with some studies [22,59] showing that the combination treatment was more effective than the use of each compound alone in controlling diabetes-linked pathologies during this period. Such findings are essential to highlight the beneficial effects of the prolonged treatment period with both metformin and resveratrol, especially since it is already known that diabetes and its related complications can induce long-term damage and failure of various organ systems [75,76]. Moreover, summarized findings report on the beneficial effects of both metformin and resveratrol in already diabetic animals, further inferring that additional studies are necessary to understand the impact of treating diabetic patients who are at increased risk of developing microvascular and macrovascular complications such as retinopathy and CVDs.

## 5. Conclusions

**Figure 2:**
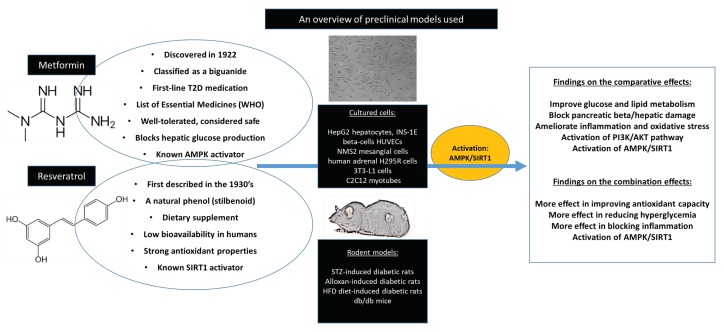
Summary of evidence on the comparative effects of metformin and resveratrol. In addition to covering the background information for each compound, a summary of preclinical models and findings reported in review are given. In brief, through various experimental models, metformin and resveratrol displayed comparative effects in ameliorating diabetes-associated complications by improving essential metabolic parameters (including glucose and lipid metabolism), blocking pancreatic/hepatic damage, as well as ameliorating both oxidative stress and inflammation. Interestingly, the combined use of these drugs could present with much better antioxidant capacity, anti-hyperglycemic effect, and attenuation of inflammation. Abbreviations: AMPK: AMP-activated protein kinase; AKT: protein kinase B; HFD: high fat diet; HUVECs: human umbilical vascular endothelial cells; PI3K: phosphatidyl-3-kinase; SIRT1: NAD-dependent deacetylase sirtuin-1STZ: streptozotocin; T2D: type 2 diabetes.

Although widely used interventions like metformin can control diabetes mellitus-related complications, the escalating prevalence of the metabolic syndrome warrants further investigation into alternative beneficial therapies. Food-derived bioactive compounds are increasingly explored for their ameliorative effects against metabolic diseases. For example, our group and others have extensively examined the beneficial effects of rooibos tea, including its bioactive compounds such as aspalathin, as well as other pharmacological compounds with abundant antioxidant properties like berries, n-acetyl cysteine, and gallic acid against diverse metabolic disorders [16,17,18,75,76,77,78,79,80]. Consistently, an extensive literature has been reviewed on the impact of resveratrol in ameliorating metabolic disease-associated complications [20,66,67]. Essentially, preclinical studies summarized in this review show that resveratrol has comparative effects in controlling diabetes-related complications as metformin. Figure 2 gives a summary of the evidence. Notably, emerging data also showed that the combination uses of these compounds could provide an even better synergistic effect than the use of each compound alone. Clinical data on the combined effect of these compounds is still scarce, however, some clinical studies support the notion that resveratrol supplementation in patients on metformin could be equally effective in managing important diabetes-related complications like blood glucose or insulin levels, as well as systolic blood pressure. However, these findings are still preliminary and confirmation is needed from well-designed clinical studies.

## Figures and Tables

**Table 1:** An overview of in vitro experimental studies reporting on the comparative impact of metformin to resveratrol on diabetes-associated complications.

Author, Year	Experimental Model, Dose Used, and Intervention Period	Experimental Outcome and Proposed Mechanism
Zang et al. 2006 [33]	HepG2 hepatocytes exposed to high glucose before treated with metformin (2 mmol/L) or resveratrol (10 μmol/L) for 1 h	Metformin and resveratrol significantly stimulated 5’ AMP-activated protein kinase (AMPK) phosphorylation and inhibited intracellular levels of triglycerides
Vetterli et al. 2011 [34]	INS-1E beta-cells and human islets were cultured with 25 μM resveratrol or 5 mM metformin for 24 h	Resveratrol improved glucose-stimulated insulin secretion. This effect was associated with elevated glycolytic flux, resulting in increased glucose oxidation, ATP generation, and mitochondrial oxygen consumption. Such changes correlated with up-regulation of key genes for β-cell function, i.e., glucose transporter (GLUT)2, glucokinase, pancreatic and duodenal homeobox 1 (PDX-1). In human islets, chronic resveratrol treatment similarly increased both the glucose secretory response and expression of the same set of genes, eventually restoring the glucose response in islets obtained from one type 2 diabetic donor. Overexpression of Sirt1 (silent mating type information regulation 2 homolog 1) in INS-1E cells potentiated resveratrol effects on insulin secretion. Conversely, inhibition of SIRT1 abolished resveratrol effects on glucose responses
Zheng et al. 2012 [35]	Bovine retinal capillary endothelial cells (BRECs) exposed to high glucose before treatment with metformin (1 mmol/L) or resveratrol (100 mmol/L) for 1 week	Metformin inhibited the increase of mitochondrial reactive oxygen species-mediated glyceraldehyde-3-phosphate dehydrogenase by poly (ADP-ribose) polymerase (PARP) activity through the upregulation of liver kinase B1/AMP-activated protein kinase (LKB1/AMPK). Metformin also performed similarly to resveratrol in suppressing nuclear factor kappa-light-chain-enhancer of activated B cells (NF-κB) and bcl-2-like protein 4 (BAX) expression. Furthermore, metformin suppressed hyperglycemia stress in the diabetic retinas, which may be involved in the SIRT1/LKB1/AMPK pathway
Choi et al. 2013 [36]	Compared seven identified caloric restriction mimetics (CRMs) which extend the lifespans of various organisms including caffeine, curcumin, dapsone, metformin, rapamycin, and resveratrol at concentrations ranging from 1 to 100 μM for different culture times in a single model, *Saccharomyces cerevisiae*	Rapamycin extended chronological lifespan (CLS), but other CRMs failed to extend CLS. Rapamycin enhanced mitochondrial function like caloric restriction did, but other CRMs did not. Both caloric restriction and rapamycin worked on mitochondrial function, but they worked at different windows of time during the chronological aging process
Kim et al. 2013 [37]	NMS2 mesangial cells exposed to high glucose before treatment with resveratrol (1, 10 or 50 ng/mL), or metformin (1 mmol/L) for 48 h	Resveratrol prevented high-glucose-induced oxidative stress and apoptosis in cultured mesangial cells through the phosphorylation of AMPK and activation of SIRT1/ peroxisome proliferator-activated receptor gamma coactivator 1 (PGC-1)α signalling and the downstream effectors, peroxisome proliferator-activated receptor (PPARα)/estrogen-related receptor (ERR)-1α/sterol regulatory element-binding protein (SREBP)1
Zhang et al. 2015 [38]	Human umbilical vascular endothelial cells (HUVECs) were exposed to high glucose for 6 days, or 3 days followed by 3 days of normal glucose treatment with or without resveratrol (0, 1.25, 2.5, 5, or 10 μM) or metformin (0, 1, 10, 50, 100, 250 μM)	Resveratrol and metformin treatment prevented senescent "memory" by modulating SIRT1/p300/p53/p21 pathway. Notably, early and continuous treatment of metformin, but not resveratrol, was particularly important for preventing senescent "memory
Li et al. 2016 [39]	Differentiated 3T3-L1 cells were incubated with metformin (1 mM) or resveratrol (10 μM) under normoxia for 4 h	Metformin and resveratrol reduced ATP production and prevented the reduction in oxygen tension in 3T3-L1 cells, suggesting that it prevented hypoxia by limiting oxygen consumption, whereas resveratrol reduced HIF-1α accumulation by promoting its proteasomal degradation via the regulation of AMPK/SIRT1
Li et al. 2016 [25]	3T3-L1 adipocytes with high glucose (33 mM) for 24 h were incubated with metformin (1 mM) or resveratrol (10 μM) under normoxia for 4 h	Metformin and resveratrol inhibited inflammation and reduced cell apoptosis in adipose tissue or adipocytes exposed to high glucose
Vasamsetti et al. 2016 [40]	THP-1 monocytes treated with metformin (0.5–2 mM) or resveratrol (0-100 μmmol/L) for 24 h	Similar to resveratrol, metformin dose-dependently increased glutathione (GSH) levels. Furthermore, incubation of cells with buthionine sulfoximine (BSO) did not affect either metformin-or resveratrol-mediated AMPK activation
Zhao et al. 2016 [42,44]	3T3-L1 cells and C2C12 myotubes were treated with metformin (1mmol/L) or resveratrol (10 μmol/L) under hypoxia conditions for 16 h	Metformin or resveratrol ameliorated insulin resistance in muscle cells by blocking free fatty acid trafficking
Marti et al. 2017 [41]	Human adrenal H295R cells were treated with metformin and resveratrol at 1 mM. for 72 h	Metformin and resveratrol was found to inhibit protein expression and enzyme activities of cytochrome enzymes (CYP17 and CYP21). It did not alter CYP17 and CYP21 mRNA expression, nor protein degradation. Only SIRT3 mRNA expression was found to be altered by resveratrol, but SIRT1, 3 and 5 overexpression did not result in a change in the steroid profile of H295R cells
Cuyàs et al. 2018 [43]	In silico analysis focusing on the molecular docking and dynamic simulation of the putative interactions between metformin and SIRT1. Using eight different crystal structures of human SIRT1 protein In vitro enzymatic assays	Metformin was predicted to interact with the very same allosteric site occupied by resveratrol and other sirtuin-activating compounds (STATCs) at the amino-terminal activation domain of SIRT1. Second, metformin was predicted to interact with the NAD+ binding site in a manner slightly different to that of SIRT1 inhibitors containing an indole ring. Third, metformin was predicted to interact with the C-terminal regulatory segment of SIRT1 bound to the NAD+ hydrolysis product ADP-ribose, a "C-pocket"-related mechanism that appears to be essential for mechanism-based activation of SIRT1 Enzymatic assays confirmed that the net biochemical effect of metformin and other biguanides such as a phenformin was to improve the catalytic efficiency of SIRT1 operating in conditions of low NAD+ in vitro
Nowak et al. 2019 [45]	Human peripheral blood CD34+ cells were isolated from peripheral blood mononuclear cells (PBMCs) obtained from a healthy volunteer cultured on day 6 with 7.5, 15, or 30 μmol/L resveratrol or 5.0 mmol/L metformin for 48 h	Failed to stabilize tubes and or enhance the paracrine angiogenic activity of human myeloid angiogenic cells

**Table 2:** An overview of in vivo experimental studies reporting on the comparative effect of metformin to resveratrol against diabetes-associated complications.

Author, Year	Experimental Model, Dose Used, and Intervention Period	Experimental Outcome and Proposed Mechanism
Chi et al. 2007 [48]	Streptozotocin (STZ)-induced diabetic Wistar rats treated with resveratrol (3 mg/kg) or metformin (100 mg/kg) for 7 days	Resveratrol produced a hypoglycemic effect in a dose-dependent manner and increased insulin levels in diabetic rats. This was followed by reduced plasma glucose and activation of phosphatidyl-3-kinase (PI3K). Resveratrol normalized hepatic phosphoenolpyruvate carboxykinase (PEPCK) expression and increased glucose transporter (GLUT)4 expression in the soleus muscle of STZ-diabetic rats. In STZ-diabetic rats, resveratrol lowered plasma glucose to a similar level as compared to the effect exerted by metformin
Bagul et al. 2012 [49]	Fructose-fed male Sprague Dawley rats administered metformin (300 mg/kg/day) or resveratrol (10 mg/kg/day) orally for 8 weeks	Although both drugs normalized altered metabolic parameters, resveratrol showed more potency in improving insulin sensitivity. Similarly, while metformin administration failed to normalize the increased thiobarbituric acid reactive substances (TBARS) levels and decreased superoxide dismutase (SOD) activity, resveratrol showed an enhanced effect in attenuating oxidative stress parameters, partially through the upregulation of nuclear factor erythroid 2-related factor 2 (NRF2)
Sun et al. 2014 [50]	Fructose-fed Sprague-Dawley rats simultaneously received resveratrol (20 mg/kg) or metformin (100 mg/kg) by oral gavage every day for 8 weeks	Long-term fructose-feeding in rats induced dysregulation of adipocytokine expression in perivascular adipose tissue (PVAT) and the loss of endothelium-dependent vasodilation, whereas oral administration of resveratrol and metformin reversed these alterations
Deng et al. 2016 [51]	Uterine-specific deletion of transformation-related protein 53 (p53d/d mice) treated with metformin orally (1 mg/kg BW per dose) on days 8, 10, and 12. Resveratrol was given (30 mg/kg BW per dose) on days 8, 10, 12, and 14	Treatment of pregnant p53d/d mice with either the antidiabetic drug metformin or the antioxidant resveratrol activated AMPK signaling and inhibited mammalian target of rapamycin complex 1 (mTORC1) signaling in decidual cells. Both metformin and resveratrol protected against spontaneous and inflammation-induced preterm birth in p53d/d mice
Kaur et al. 2016 [52]	STZ-induced diabetic rats were fed raw garlic homogenate (250 mg/kg/day), resveratrol (25 mg/kg/day), and metformin (500 mg/kg/day) orally for 4 weeks	Administration of garlic, resveratrol, and metformin significantly normalized altered metabolic and oxidative stress parameters as well as histopathological changes. Treatment with these compounds also ameliorated pancreatic β-cell damage and hepatic injury
Li et al. 2016 [39]	ICR male mice fed high-fat diet before treatment with metformin (200 mg/kg), resveratrol (50 mg/kg) or TUDCA (50 mg/kg) by gavage every day for 7 days	Metformin or resveratrol comparably prevented hypoxia and reduced hypoxia-inducible factor 1 (HIF-1)α accumulation with dephosphorylation of inositol-requiring enzyme 1α and eukaryotic initiation factor 2α, indicative of suppression of hypoxic HIF-1α activation and endoplasmic reticulum stress. Metformin and resveratrol down-regulated the expression genes related collagen deposition such as elastin and lysyl oxidase. The increased gene expressions of tumor necrosis factor (TNF)-α, interleukin (IL)-6, monocyte chemoattractant protein 1 and F4/80 were also down-regulated by metformin and resveratrol
Li et al. 2016 [25]	STZ-diabetic ICR male mice and followed by oral administration of metformin (200 mg/kg), resveratrol (50 mg/kg) or ER stress inhibitor TUDCA (50 mg/kg) for 7 days	Metformin and resveratrol inhibited reactive oxygen species (ROS)-associated mitochondrial fission by upregulating Drp1 phosphorylation (Ser 637) in an AMPK-dependent manner, and then suppressed endoplasmic reticulum (ER) stress indicated by dephosphorylation of endoribonuclease 1α and eukaryotic initiation factor 2 in the adipose tissue
Reddy et al. 2016 [53]	Sprague-Dawley rats on high-fructose diet received resveratrol and metformin together with fructose diet at a single dose of 10 mg/kg/day of resveratrol orally or 300 mg/kg/day of metformin orally, for 8 weeks	Resveratrol was more effective in protecting both the metabolic (prediabetic) and affective (anxiety) disorders than metformin. Molecular studies showed that recovery was associated with the upregulation of few nuclear sirtuins that act epigenetically—SIRT1 and 7
Vilar-Pereira et al. 2016 [54]	BALB/c mice infected type I Colombian strain of *T. cruzi* before treatment with 15 mg/kg trans-resveratrol, 40 mg/kg resveratrol and 500 mg.kg^-1^ metformin	Resveratrol increased heart rates and reversed sinus arrhythmia, atrial and atrioventricular conduction disorders; restored a normal left ventricular ejection fraction, improved stroke volume and cardiac output. Resveratrol activated the AMPK-pathway and reduced both ROS production and heart parasite burden, without interfering with vascularization or myocarditis intensity. Metformin and tempol mimicked the beneficial effects of resveratrol on heart function and decreased lipid peroxidation, but did not alter parasite burden
Zhao et al. 2016 [42,44]	High fat diet-fed ICR mice received metformin (200 mg/kg), or resveratrol (50 mg/kg), every day for 10 days	Metformin and resveratrol attenuated adipose hypoxia, inhibited HIF-1α expression and inflammation in the adipose tissue. Metformin and resveratrol inhibited lipolysis. Metformin and resveratrol reduced free fatty acid influx and diacylglycerol accumulation and thus improved insulin signaling in the muscle by inhibiting protein kinase C (PKC)θ translocation
Barger et al. 2017 [55]	White adipose tissue, gastrocnemius muscle, heart, and brain neocortex from seven mouse strains (C3H/HeJ, CBA/J, DBA/2J, B6C3F1/J, 129S1/SvImJ, C57BL/6J, and BALB/cJ) treated with trans-resveratrol (510 mg/kg) or metformin (1909 mg/kg) supplemented in diet for 3 months	Metformin and resveratrol were effective as the caloric restriction in reducing plasma interferon-gamma levels
Stockinger et al. 2017 [56]	Male C57BL/6J wild-type mice fed 40% less fat calories starting at 4 months of age containing resveratrol at 400 mg/kg or metformin at 1,000 mg/kg starting at 1 year of age. Mice were sacrificed at 2 years of age to examine NMJs and muscle fibers	Resveratrol significantly slowed aging of neuromuscular junctions (NMJs) in the extensor digitorum longus muscle of 2-year-old mice. Resveratrol also preserved the morphology of muscle fibers in old mice. Although metformin slowed the rate of muscle fiber aging, it did not significantly affect aging of NMJs. Resveratrol also increased the number of postsynaptic sites on myotubes exhibiting a youthful architecture, suggesting that resveratrol directly affects the NMJ
Mehdi et al. 2018 [26]	C57BL/6 J male mice received 400 mg/kg of resveratrol or metformin 250 mg/kg, via gavage for 45 days	In brown adipose tissue, resveratrol significantly reduced the lipid droplet-associated (PLIN5) protein level and gene expression. In heart tissue, resveratrol, and strength training, decreased the plin5 expression, but metformin increased the gene expression. In skeletal muscle, resveratrol, strength training, cold and metformin significantly increased the plin5 expression at the gene and protein level
Rehman et al. 2018 [57]	Alloxan-induced albino rats received metformin (500 mg/kg/day) or resveratrol (30 mg/kg/day) for 30 days	Resveratrol alone and/or in combination with vitamin E exhibited a highly significant therapeutic potential by ameliorating the glycemia-induced modulations. Moreover, resveratrol in combination with vitamin E also exhibited a better therapeutic effects when compared with that of metformin

**Table 3:** An overview of experimental studies reporting on the combination use of metformin with resveratrol against diabetes-associated complications.

Author, Year	Experimental Model, Dose Used, and Intervention Period	Experimental Outcome and Proposed Mechanism
Bruckbauer et al. 2013 [58]	C2C12 skeletal myotubes and 3T3-L1 adipocytes treated with resveratrol 0.2 μM, hydroxymethylbutyrate (HMB) 5 μM, and metformin 0.1 mM alone or in combination. Type 2 diabetic (db/db) mice treated for 2 weeks with high (1.5 g/kg diet), low (0.75 g/kg diet), or very low (0.25 g/kg diet) doses of metformin	The combination of metformin-resveratrol-HMB significantly increased fat oxidation, AMP-activated protein kinase (AMPK), and SIRT1 activity in muscle cells compared with metformin or resveratrol-HMB alone. A similar trend was found in 3T3L1 adipocytes In mice, the two lower doses of metformin exerted no independent effect but, when combined with resveratrol-HMB, both low-dose and very low-dose metformin improved insulin sensitivity (HOMA(IR)), plasma insulin levels, and insulin tolerance test response to a level comparable with that found for high-dose metformin. Additionally, the metformin-resveratrol-HMB combination decreased visceral fat and liver weight in mice
Fu et al. 2015 [59]	High fat diet-fed (HFD) male C57BL/6 mice received a combination of leucine (24 g/kg diet), resveratrol (12.5 mg/kg/diet) and metformin (0.05–0.5 g/kg diet) for 6 weeks	The combination of leucine, metformin, and resveratrol was more effective than the use of metformin as a monotherapy in improving glucose tolerance
Duarte-Vázquez et al. 2016 [22]	Type 2 diabetic (db/db) mice received resveratrol (20 mg/kg/day), metformin (150 mg/kg/day) and combined metformin/resveratrol therapy for 5 weeks	Data clearly showed that combined metformin/resveratrol treatment reduced obesity, glucose, and triglyceride levels, as well as improving renal function and partially improving liver function in diabetic mice
Frendo-Cumbo et al. 2016 [23]	Male C57BL6 mice fed HFD were given metformin (231 mg/kg/day), resveratrol (93 mg/kg/day), or combined (metformin 232.01 ± 17.12 mg/kg/day and resveratrol 92 mg/kg/day) treatment groups for 4 weeks	Treatment with each compound alone did not have beneficial effects on glucose tolerance, although metformin significantly improved insulin tolerance. Glucose and insulin tolerance were significantly improved in the combination treatment. This was mirrored by enhanced insulin-stimulated protein kinase B (AKT) phosphorylation in triceps muscle and inguinal subcutaneous adipose tissue. However, improvements with combination treatment did not affect body weight, adiposity, or markers of adipose tissue inflammation
Furat et al. 2018 [60]	Dehydroepiandrosterone (DHEA)-induced Wistar rats were given resveratrol (20 mg/kg/day), metformin (300 mg/kg/day) and combined therapy for 28 days	Metformin and combined treatments reduced the body and ovary weights. All the treatment groups decreased luteinizing hormone, follicle-stimulating hormone, tumor necrosis factor (TNF)-α and tissue anti-mullerian hormone(AMH) levels, whereas metformin was unable to improve the increased malondialdehyde (MDA) and plasma AMH levels. Resveratrol and metformin increased SIRT1 and AMPK immunoreactivity
Das et al. 2019 [61]	Wistar fed HFD received metformin (0.5 gm/kg), resveratrol (5, 10, and 20 mg/kg), or the combination of a half dose of metformin and resveratrol (10 mg/kg) for 2 weeks	The combination treatment significantly decreased fasting blood glucose. This effect was consistent with significant improvement in dyslipidemia compared to their baseline values. There was also a significant change in the serum MDA level and superoxide dismutase activity
Yang et al. 2019 [62]	Male C57BL/6J mice fed HFD treated with/without metformin (250 mg/kg/day) and resveratrol (100 mg/kg/day), or the combination of metformin (250 mg/kg/day and 100 mg/kg/day, respectively) for 5 weeks	Resveratrol alone or when combined to metformin promoted phosphorylation of cortex AMPK and raptor (the regulatory subunit of mTORC1). Furthermore, all treatment reduced p62 content; while activating mTOR and Unc-51 like autophagy activating kinase (ULK1) in the cortex and hippocampus. Brain-derived neurotropic factor (BDNF) was significantly decreased resveratrol or combination treatment

## Abbreviations

AMPK: AMP-activated protein kinase; AKT: protein kinase B; BAX: bcl-2-like protein 4; CVDs: cardiovascular diseases; CYPs: cytochromes P450 enzymes; GSH: glutathione; HFD: high fat diet; HUVECs: human umbilical vascular endothelial cells; IL: interleukin; mTORC1: mammalian target of rapamycin complex 1; NF-κB: nuclear factor kappa-light-chain-enhancer of activated B cells; NMJs: neuromuscular junctions; PEPCK: phosphoenolpyruvate carboxykinase; PI3K: phosphatidyl-3-kinase; PRISMA: Preferred Reporting Items for Systematic reviews and Meta-Analysis; ROS: reactive oxygen species; SIRT1: NAD-dependent deacetylase sirtuin-1; SOD: superoxide dismutase; STZ: streptozotocin; T1D: type 1 diabetes; T2D: type 2 diabetes; TNF-α: tumor necrosis factor alpha.
